# Static and dynamic functional connectivity of the bilateral insula in patients with migraine without aura: a resting-state fMRI study

**DOI:** 10.3389/fneur.2026.1898658

**Published:** 2026-08-31

**Authors:** Guo-Yun Liu, Zi-Min Cao, Yi-Chao Chen, Ling-Hua Li, Chao-Qun Yan, Jun Wang

**Affiliations:** 1 Department of Acupuncture and Moxibustion, Dongzhimen Hospital, Beijing University of Chinese Medicine, Beijing, China; 2 Department of Acupuncture and Massage, Shanghai General Hospital, Shanghai Jiao Tong University School of Medicine, Shanghai, China

**Keywords:** dynamic functional connectivity, functional magnetic resonance imaging, insula, migraine without aura, resting state functional connectivity

## Abstract

**Objective:**

To investigate alterations in resting-state functional connectivity (rs-FC) and the standard deviation of dynamic functional connectivity (dFCstd) of the bilateral insula in patients with migraine without aura (MwoA), and to examine their associations with clinical symptoms.

**Methods:**

Resting-state functional magnetic resonance imaging (rs-fMRI) was conducted on 45 patients with MwoA and 45 healthy controls (HCs). The left and right insulae were defined as seed regions according to the AAL116 atlas. rs-FC and dFCstd were calculated between each insular seed and the remaining 115 brain regions, with dFCstd defined as the standard deviation of Fisher-z transformed connectivity estimates across sliding windows. Group differences were evaluated with general linear models adjusted for age and sex, with false discovery rate (FDR) correction applied for multiple comparisons. Associations between altered functional connectivity and clinical measures were further examined within the MwoA group.

**Results:**

Patients with MwoA had higher rs-FC than HCs for the left and right insulae with the right hippocampus. Dynamic connectivity analysis identified lower dFCstd involving the left insula and ipsilateral caudate nucleus and higher dFCstd involving the left insula and ipsilateral superior frontal gyrus, orbital part. All group differences survived FDR correction. Exploratory correlation analyses showed that several altered connectivity measures were associated with monthly migraine attack frequency, visual analog scale (VAS) scores, and Self-Rating Depression Scale (SDS) standard scores.

**Conclusion:**

Combined rs-FC and dFCstd analyses revealed altered functional connectivity involving the insula in patients with MwoA. These connectivity patterns may reflect disturbances in neural circuits supporting pain processing, emotion regulation, and cognitive control, providing complementary imaging evidence for brain network dysfunction in MwoA.

## 1 Introduction

Migraine is a prevalent chronic neurological condition marked by recurrent headaches of moderate to severe intensity. Attacks are commonly accompanied by nausea, vomiting, photophobia, and phonophobia, and many patients also experience emotional symptoms or psychiatric comorbidities (1). The Global Burden of Disease 2021 study indicated that around 1.1 billion people worldwide are affected by migraine, with a prevalence of 15.2%. Migraine is also a leading cause of years lived with disability (YLDs) worldwide (2–4). In addition to impairing quality of life, migraine creates a considerable social and economic burden (5). With advances in neuroimaging, migraine is increasingly viewed not solely as a vascular disorder but as a brain network disorder involving multiple systems related to pain perception, sensory modulation, affective memory, and cognitive control (6–8). Investigating abnormalities in brain functional connectivity may therefore help clarify the neuropathological mechanisms underlying migraine.

Migraine is characterized by recurrent attacks and cyclical fluctuations. Brain functional connectivity may also vary across the interictal, preictal, ictal, and postictal phases of migraine (8). The interictal period accounts for a substantial proportion of the disease course. Even during this period, patients may show pain anticipation, sensory sensitization, and impaired emotion regulation (9–11). Imaging during the interictal phase allows brain function to be examined in the absence of an ongoing headache attack, thereby helping to characterize functional features associated with the interictal state.

Resting-state functional magnetic resonance imaging (rs-fMRI) enables noninvasive characterization of intrinsic brain activity and interregional functional coupling. Conventional resting-state functional connectivity (rs-FC) summarizes temporal correlations between regional blood-oxygen-level-dependent (BOLD) signals across the entire scan but may not capture fluctuations in connectivity over shorter time intervals. However, whether functional connectivity shows altered temporal variability within a single resting-state scan during the interictal period remains unclear. Within-scan temporal variability describes the extent to which functional connectivity fluctuates around its mean and may reveal abnormalities in dynamic interregional coordination that are obscured by scan-averaged connectivity. Dynamic functional connectivity (dFC) provides a complementary approach by characterizing time-varying connectivity patterns. The standard deviation of dFC across sliding windows (dFCstd) quantifies temporal fluctuations in functional connectivity within a scanning session (12). dFC methods have been increasingly applied in migraine research in recent years (13, 14). Combining rs-FC with dFCstd may therefore provide a more comprehensive view of brain dysfunction in migraine by capturing both average connectivity strength and temporal variability.

The insula contributes to pain perception, interoceptive processing, and emotional experience, and supports the integration of sensory, affective, cognitive, and autonomic information (15). Evidence from pain research has consistently emphasized the insula as a key node in pain processing, suggesting that dysfunction of this region may disrupt the coordination of nociceptive perception, interoceptive signals, and emotional responses (16, 17). In migraine, altered insular functional connectivity has been implicated in abnormal processing of pain and internal or external sensory stimuli (18). The integrative role of the insula is closely related to common interictal features of migraine, including sensory hypersensitivity, altered interoception, pain anticipation, and emotional symptoms, making the insula particularly relevant for investigating functional connectivity features that remain detectable between attacks. Research examining insular subregions in migraine without aura (MwoA) has revealed both structural abnormalities and altered functional connectivity. Some of these insula-related changes have also shown clinical relevance, with associations reported for migraine duration, attack burden, and headache severity (19). In addition, ictal resting-state fMRI research has revealed connectivity changes involving the sensorimotor network and posterior insula, with these changes related to attack duration (20). This evidence highlights the insula as a relevant node for investigating brain network dysfunction in migraine. Although the insula is functionally heterogeneous, migraine-related processing may involve multiple insular regions and their associated networks. Because the present study aimed to characterize the insula as an integrated network node rather than to test subregion-specific effects, the entire left and right insulae were selected as seed regions.

Although insular functional connectivity has been increasingly investigated in migraine, most existing work has focused on static connectivity patterns of insular subregions or functional connectivity changes during migraine attacks. The temporal variability of insular connectivity during the interictal period remains less well characterized. We focused specifically on MwoA because it is the most prevalent migraine subtype and provides a relatively homogeneous clinical context for examining interictal insula-centered connectivity abnormalities, while reducing potential heterogeneity associated with aura-related cortical events and transient visual or sensory symptoms. Therefore, using the bilateral insulae as seed regions, this study combined rs-FC and dFCstd to compare average connectivity strength and temporal variability between patients with MwoA and healthy controls (HCs). Associations between altered connectivity measures and clinical symptoms were further examined to assess the clinical relevance of these connectivity abnormalities.

## 2 Materials and methods

### 2.1 Participants

A total of 45 right-handed patients with MwoA were recruited from the outpatient clinic of Dongzhimen Hospital, Beijing University of Chinese Medicine. Eligible patients with MwoA met the following criteria: (1) age 18–65 years; (2) MwoA diagnosis based on the International Classification of Headache Disorders, 3rd edition (ICHD-3) (21); (3) absence of migraine attacks within the 72 h preceding MRI scanning; (4) age at first onset ≤ 50 years; (5) migraine duration ≥1 year; and (6) no preventive treatment for migraine, including oral or injectable therapy, within the preceding month. The exclusion criteria were as follows: (1) more than 10 headache attacks per month; (2) use of preventive medications, antiepileptic drugs, or antidepressants within the preceding month; (3) pregnancy or lactation; (4) other types of headache or secondary headache; (5) drug or alcohol abuse or dependence; and (6) contraindications to MRI or inability to complete the scan.

During the same period, 45 right-handed HCs were recruited to ensure a similar age and sex distribution across groups. All HCs had no history of migraine or other primary or secondary headaches, psychiatric or neurological disorders, chronic systemic diseases, drug or alcohol abuse or dependence, pregnancy or lactation, participation in other clinical trials, or contraindications to MRI.

The study protocol was approved by the Ethics Committee of Dongzhimen Hospital, Beijing University of Chinese Medicine (approval No. 2022DZMEC-176-02). All participants provided written informed consent before enrollment.

### 2.2 Clinical assessments

Demographic information and psychological status were assessed in all participants, including age, sex, Self-Rating Depression Scale (SDS) scores, and Self-Rating Anxiety Scale (SAS) scores. Patients with MwoA underwent additional migraine-related clinical assessments, including the Migraine-Specific Quality of Life Questionnaire (MSQ), with its emotional function (MSQ-E), role function-restrictive (MSQ-R), and role function-preventive (MSQ-P) domains and total score; visual analog scale (VAS) scores for pain intensity; monthly migraine attack frequency, defined as the number of migraine attacks per month; and monthly analgesic use. Acute rescue medications were permitted when clinically necessary, and their use was recorded as monthly analgesic use. No prespecified medication-free interval was required before MRI scanning. All scales were scored according to their standard scoring instructions.

### 2.3 rs-fMRI data acquisition

MRI scanning was conducted at the Department of Radiology, Beijing Hospital of Traditional Chinese Medicine, Capital Medical University, using a Siemens 3.0 T Skyra MRI scanner (Siemens, Erlangen, Germany). During scanning, participants lay in the supine position and were asked to keep still, remain awake and relaxed, avoid deliberate mental activity, and reduce head movement as much as possible.

Resting-state functional images were obtained with an axial gradient-echo echo-planar imaging (GRE-EPI) sequence. The acquisition parameters were as follows: repetition time (TR) = 2,000 ms, echo time (TE) = 30 ms, field of view (FOV) = 224 mm × 224 mm, flip angle = 90°, slice thickness = 3.5 mm, voxel size = 3.5 mm × 3.5 mm × 3.5 mm, bandwidth = 2,368 Hz, and 32 slices. The resting-state scan was 8 min in duration and comprised 240 volumes.

### 2.4 fMRI data preprocessing

Preprocessing was carried out with DPARSF and SPM12 in MATLAB R2021a. To minimize the influence of unstable signals at the beginning of the scan, the initial 10 volumes were discarded, leaving 230 volumes for further analysis. The remaining images were adjusted for slice acquisition timing and realigned for head motion. They were then transformed into Montreal Neurological Institute space and resampled to 3 mm × 3 mm × 3 mm voxels. Spatial smoothing was performed with a Gaussian kernel of 6 mm full width at half maximum. Linear trends were removed, and nuisance covariates, including the Friston 24-parameter head-motion model, white matter signal, and cerebrospinal fluid signal, were regressed out. Mean framewise displacement was calculated for each participant using the Power method. Scrubbing or frame censoring was not performed, and no FD threshold was used to censor individual volumes. A temporal bandpass filter of 0.01 to 0.08 Hz was applied to retain low frequency BOLD fluctuations and reduce slow drift and high frequency noise. Participants were excluded when head motion exceeded 3 mm of translation or 3° of rotation, or if image quality was insufficient. After preprocessing, regional mean time series were extracted according to the AAL116 atlas and used for subsequent rs-FC and dFCstd analyses.

### 2.5 rs-FC calculation

The mean resting-state BOLD time series of 116 brain regions were extracted for each participant in the AAL116 atlas, and the left and right insulae were selected as seed regions. For each insular seed, its mean time series was correlated with the time series from each of the remaining 115 target regions, yielding rs-FC values for each seed and target region pair. Before statistical analysis, Pearson r values were converted to Fisher z values to improve their distributional properties. These transformed values were then used in subsequent analyses. rs-FC was used to index the average strength of functional coupling between brain regions over the whole resting state scan.

### 2.6 dFCstd calculation

dFC was quantified by applying a sliding window approach to the AAL116 regional time series. In the primary analysis, the sliding window comprised 50 TRs and moved across the time series in 1 TR increments, consistent with previous dFC studies (22). Given a repetition time of 2 s, the 50-TR window corresponded to 100 s and covered one full cycle of the lowest retained frequency of 0.01 Hz. This window length was selected to reduce spurious fluctuations associated with windows shorter than the longest retained wavelength, while considering the trade-off between correlation stability and temporal resolution (23, 24). Within each time window, the signal from each insular seed was correlated with signals from the other 115 regions using Pearson correlation analysis. The resulting correlations were transformed into Fisher *z*-values. For each seed to target connection, dFCstd was defined as the standard deviation of Fisher z values across all sliding windows. Higher dFCstd values indicated a wider range of temporal fluctuations in connectivity, whereas lower values indicated a narrower range of temporal fluctuations.

**Figure 1:**
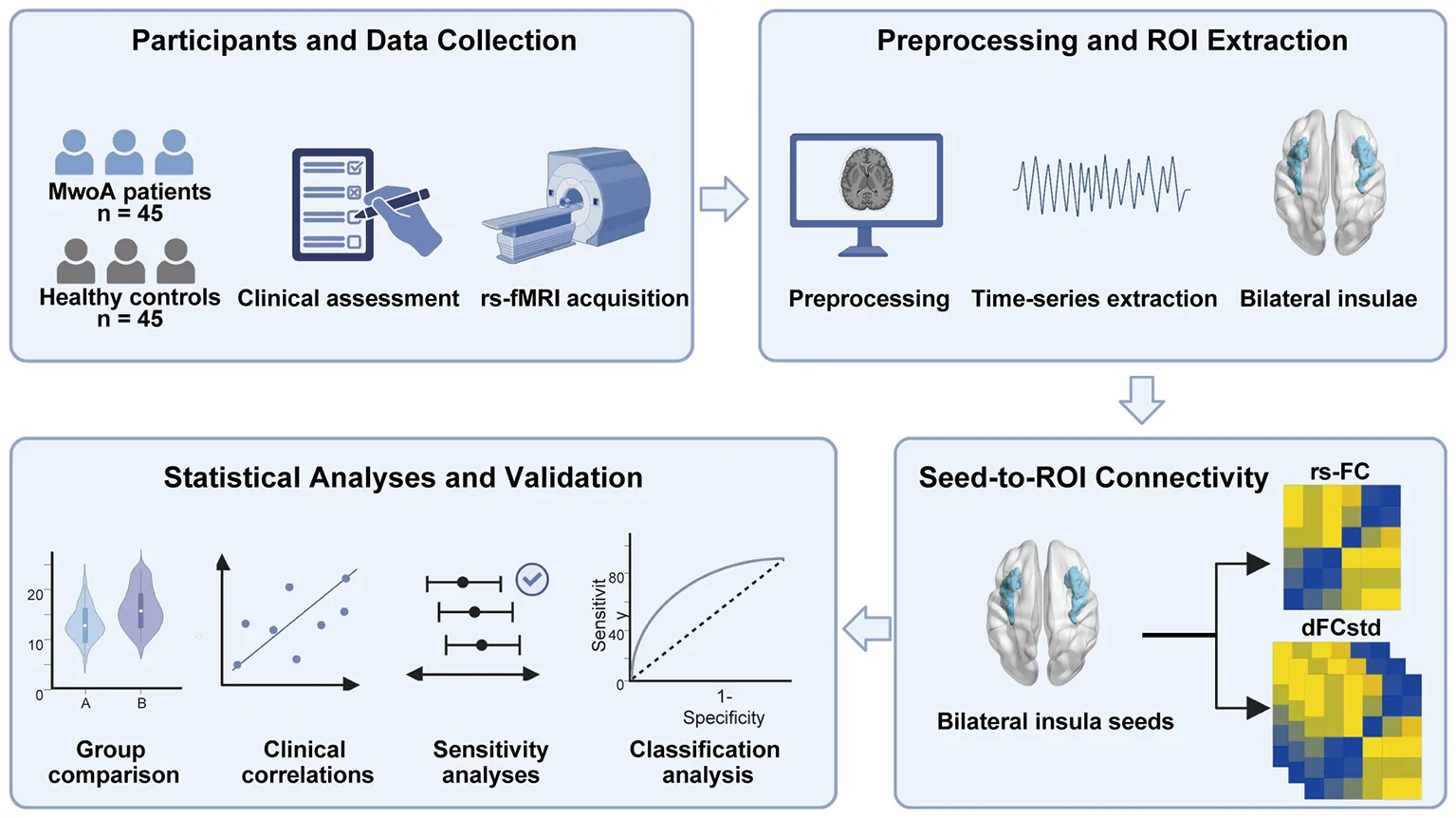
Study design and analysis workflow. Overview of participant recruitment, rs-fMRI preprocessing, bilateral insula seed-based connectivity calculation, and statistical analyses. MwoA, migraine without aura; HC, healthy controls; rs-fMRI, resting-state functional magnetic resonance imaging; ROI, region of interest; rs-FC, resting-state functional connectivity; dFCstd, standard deviation of dynamic functional connectivity. Created in BioRender. Liu, G. (2026) https://BioRender.com/19il7mc.

The overall study design and analysis workflow are shown in Figure 1.

### 2.7 Statistical analysis

#### 2.7.1 Demographic and clinical measures

Demographic, clinical, and head-motion data were analyzed using R 4.5.1. Continuous variables are presented as mean ± standard deviation and were compared using independent samples *t*-tests. Categorical variables are reported as frequencies and percentages, with group differences assessed using χ^2^ tests or Fisher's exact tests, as appropriate. Statistical significance was defined as a two sided *P*-value < 0.05.

#### 2.7.2 Group comparisons of rs-FC and dFCstd

Imaging statistical analyses were performed using custom scripts in Python 3.13. General linear models were used to compare rs-FC and dFCstd between patients with MwoA and HCs, adjusting for age and sex. Given that the bilateral insulae were defined a priori as seed regions and all analyses were conducted at the ROI-based seed-to-target level, false discovery rate (FDR) correction was applied separately at the seed level for rs-FC and dFCstd. Specifically, for each imaging measure, FDR correction was performed across the 115 connections between each insular seed and the remaining target regions. FDR-corrected *P-*values and Cohen's d were reported, with FDR-corrected *P* < 0.05 considered statistically significant.

#### 2.7.3 Clinical correlation analyses

To further evaluate the relationships between altered functional connectivity and clinical symptoms, rs-FC and dFCstd values showing significant group differences after FDR correction were extracted in the MwoA group and entered into partial correlation analyses, with age and sex controlled as covariates. The clinical measures included VAS scores, monthly migraine attack frequency, monthly analgesic use, MSQ-E, MSQ-P, MSQ-R, total MSQ scores, SAS standard scores, and SDS standard scores. Given the exploratory nature of these analyses, correlations with uncorrected *P* < 0.05 were reported as exploratory findings. The FDR correction was applied jointly across all partial correlation analyses between the functional connectivity measures showing significant group differences and the prespecified clinical variables to control for multiple comparisons.

### 2.8 Sensitivity analyses

Given the potential influence of anxiety and depressive symptoms on functional connectivity measures, additional covariate sensitivity analyses were performed. The main rs-FC and dFCstd connections showing significant group differences after FDR correction were entered as dependent variables in general linear models, with group as the independent variable. In addition to age and sex, SAS and SDS standard scores were included as covariates to evaluate the robustness of the main group differences. The *P-*values for the group effects across these connections were adjusted using FDR correction.

To assess the potential influence of residual head motion, a separate sensitivity analysis was performed for the four main altered connections. General linear models were constructed with group as the independent variable and age, sex, and mean FD as covariates. FDR correction was applied across the group effects of the four connections.

To further evaluate whether the main findings were dependent on the correction scope, we performed an additional conservative joint FDR correction across all bilateral insula seed-to-target rs-FC and dFCstd tests, including 460 comparisons in total. This sensitivity analysis evaluated the robustness of the primary seed-level FDR findings.

Because dFCstd may be affected by sliding-window length, dFCstd was additionally recalculated using window lengths of 30 TRs and 60 TRs, in addition to the 50 TR window used in the main analysis. The direction and statistical significance of the main dFCstd findings were then compared across different window lengths. Detailed procedures are provided in the Supplementary materials.

### 2.9 Exploratory classification analyses

To further evaluate the within-sample discriminative performance of the main altered insula-related connections in distinguishing patients with MwoA from HCs, exploratory classification analyses were performed. Classification features were restricted to insula-related rs-FC and dFCstd connections that showed significant group differences after FDR correction. Logistic regression and linear support vector machine models were constructed separately. Their performance was assessed through stratified fivefold cross validation. To further assess the sensitivity of performance estimates to random data partitioning, we additionally repeated the stratified fivefold cross-validation procedure 20 times using different random partitions while preserving the class distribution within each fold. Classification metrics from the repeated cross-validation were summarized as mean and standard deviation. Discriminative ability was summarized by the area under the receiver operating characteristic curve (AUC), together with accuracy, sensitivity, and specificity. Permutation testing with 5,000 label permutations was further performed to assess whether classification performance exceeded chance level.

Because these classification features were selected from group differences identified in the same sample, potential feature selection bias could not be excluded. Therefore, an additional sensitivity classification analysis was performed using all candidate connections, including 460 bilateral insula rs-FC and dFCstd connections. Within each training fold, residualization for age and sex, feature selection, standardization, and model training were performed independently. Further methodological details are described in the Supplementary materials.

## 3 Results

### 3.1 Participant characteristics

Ninety participants were included in the final analysis, including 45 individuals with MwoA and 45 HCs. Among patients with MwoA, 10 were male and 35 were female, with a mean age of 34.96 ± 12.27 years. The HC group included 17 male and 28 female participants, with a mean age of 37.67 ± 15.67 years. No significant between-group differences were found in age, sex distribution, or mean FD (all *P* > 0.05). Compared with HCs, patients with MwoA showed higher SAS and SDS standard scores. Demographic and clinical data are presented in Table 1.

**Table 1:** Demographic and clinical characteristics of participants.

Characteristics	MwoA (*n =* 45)	HCs (*n =* 45)	Statistic	*P*-value
General characteristics				
Age, mean (SD), years	34.96 (12.27)	37.67 (15.67)	*t* = −0.914	0.363
**Sex**, ***n***			χ^2^ = 2.593	0.107
Female	35	28		
Male	10	17		
Handedness (Right/Left), *n*	45/0	45/0	—	—
Mean FD, mean (SD), mm	0.160 (0.070)	0.168 (0.089)	t = −0.455	0.650
Migraine-related clinical characteristics				
MSQ-E score, mean (SD)	61.63 (27.93)	—	—	—
MSQ-P score, mean (SD)	65.27 (18.47)	—	—	—
MSQ-R score, mean (SD)	57.46 (13.87)	—	—	—
Total MSQ score, mean (SD)	61.45 (17.79)	—	—	—
VAS score, mean (SD)	6.73 (1.18)	—	—	—
Monthly migraine attack frequency, mean (SD), attacks/month	3.62 (1.61)	—	—	—
Monthly analgesic use, mean (SD), times/month	2.40 (2.11)	—	—	—
Psychological characteristics				
SAS standard score, mean (SD)	44.47 (9.50)	35.53 (7.12)	t = 5.047	< 0.001
SDS standard score, mean (SD)	43.13 (10.50)	36.31 (7.97)	t = 3.472	0.001

Values are presented as mean (SD) unless otherwise indicated. MwoA, migraine without aura; HCs, healthy controls; FD, framewise displacement; MSQ, Migraine-Specific Quality of Life Questionnaire; VAS, visual analog scale; SAS, Self-Rating Anxiety Scale; SDS, Self-Rating Depression Scale.

### 3.2 Group differences in rs-FC

**Figure 2:**
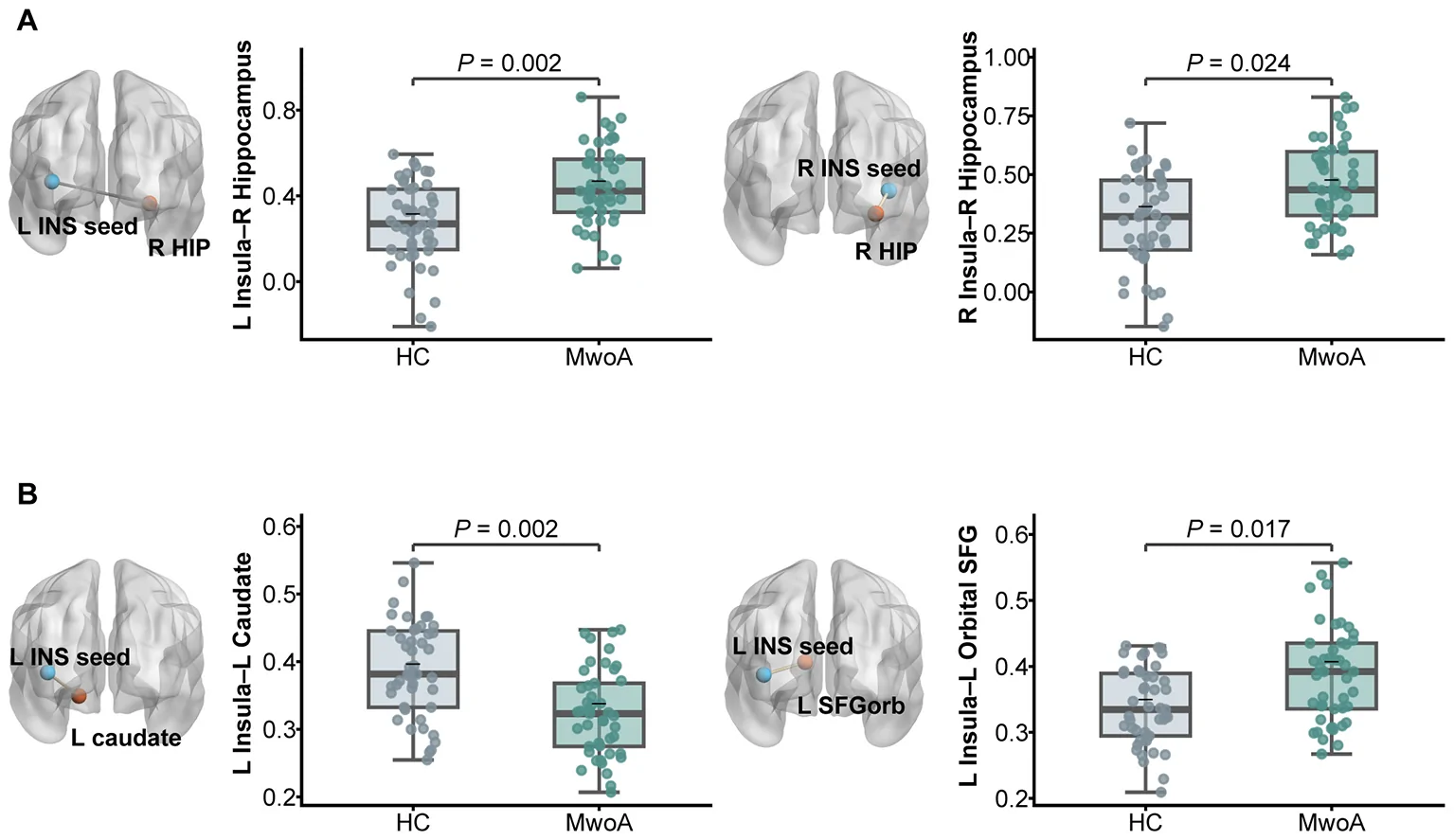
Group differences in insula-related rs-FC and dFCstd. **(A)** rs-FC differences involving the left or right insula and the right hippocampus. **(B)** dFCstd differences involving the left insula with the left caudate nucleus or the left superior frontal gyrus, orbital part. For each panel, brain renderings show the seed and target regions, and boxplots show individual connectivity values. *P*-values are FDR-corrected. MwoA, migraine without aura; HCs, healthy controls; rs-FC, resting-state functional connectivity; dFCstd, standard deviation of dynamic functional connectivity; INS, insula; HIP, hippocampus; SFGorb, superior frontal gyrus, orbital part.

Following FDR correction, higher rs-FC was observed in patients with MwoA than in HCs for the connection between the left insula and right hippocampus (*P* = 0.002, Cohen's *d* = 0.92) and the connection between the right insula and right hippocampus (*P* = 0.024, Cohen's *d* = 0.81). The rs-FC findings are detailed in Table 2 and graphically displayed in Figure 2A.

**Table 2:** rs-FC differences between the MwoA and HCs.

Seed	Target	MwoA	HCs	*t*-value	FDR-corrected *P*	Joint FDR-corrected *P*	Cohen's *d*	Direction
Insula_L	Hippocampus_R	0.446 ± 0.184	0.271 ± 0.194	4.518	0.002	0.005	0.92	MwoA > HCs
Insula_R	Hippocampus_R	0.462 ± 0.176	0.306 ± 0.205	3.870	0.024	0.032	0.81	MwoA > HCs

Values are presented as mean ± SD. Insula_L, left insula; Insula_R, right insula; Hippocampus_R, right hippocampus; rs-FC, resting-state functional connectivity; MwoA, migraine without aura; HCs, healthy controls; FDR, false discovery rate.

### 3.3 Group differences in dFCstd

**Figure 3:**
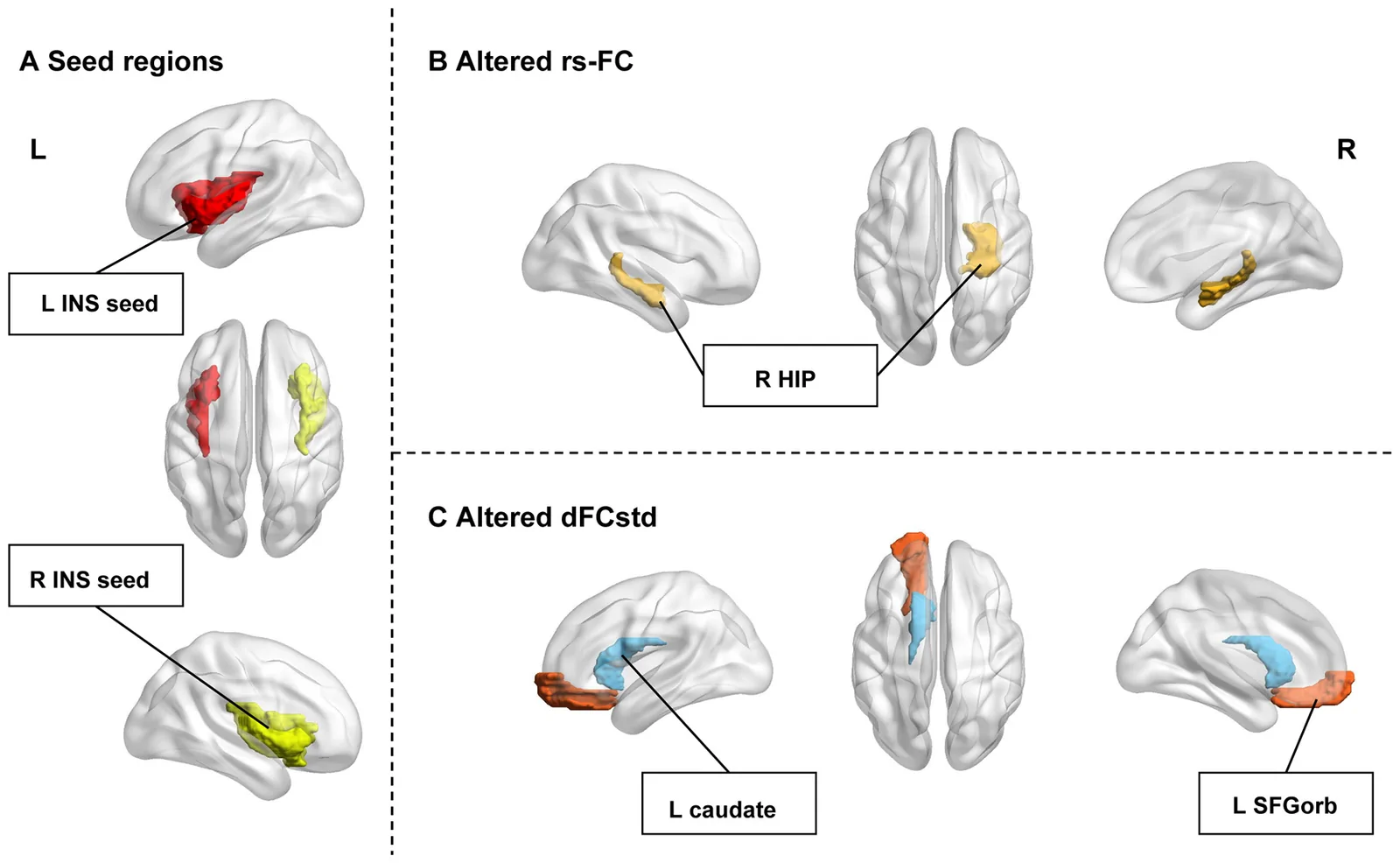
Seed regions and altered connectivity targets. Brain visualization of bilateral insula seeds and target regions showing altered rs-FC and dFCstd in patients with migraine without aura. **(A)** Bilateral insula seed regions. **(B)** Target region with altered rs-FC. **(C)** Target regions with altered dFCstd. INS, insula; HIP, hippocampus; SFGorb, superior frontal gyrus, orbital part; rs-FC, resting-state functional connectivity; dFCstd, standard deviation of dynamic functional connectivity; L, left; R, right.

For dFCstd, group comparisons after FDR correction revealed two significant alterations related to the left insular seed. Relative to HCs, the MwoA group showed reduced dFCstd of the left insular seed with the ipsilateral caudate nucleus (*P* = 0.002, Cohen's *d* = −0.98). In contrast, dFCstd of the same seed with the orbital part of the left superior frontal gyrus was increased (*P* = 0.017, Cohen's *d* = 0.73). No dFCstd differences involving the right insula survived FDR correction. The dFCstd group differences are presented in Table 3 and shown in Figure 2B. The bilateral insular seeds and altered target regions are displayed in Figure 3.

**Table 3:** dFCstd differences between the MwoA and HCs.

Seed	Target	MwoA	HCs	*t-*value	FDR-corrected *P*	Joint FDR-corrected *P*	Cohen's *d*	Direction
Insula_L	Caudate_L	0.324 ± 0.064	0.390 ± 0.070	−4.607	0.002	0.005	−0.98	MwoA < HCs
Insula_L	Frontal_Sup_Orb_L	0.387 ± 0.073	0.339 ± 0.058	3.776	0.017	0.034	0.73	MwoA > HCs

Values are presented as mean ± SD. Insula_L, left insula; Caudate_L, left caudate nucleus; Frontal_Sup_Orb_L, left superior frontal gyrus, orbital part; dFCstd, standard deviation of dynamic functional connectivity; MwoA, migraine without aura; HCs, healthy controls; FDR, false discovery rate.

### 3.4 Clinical associations of altered connectivity

**Figure 4:**
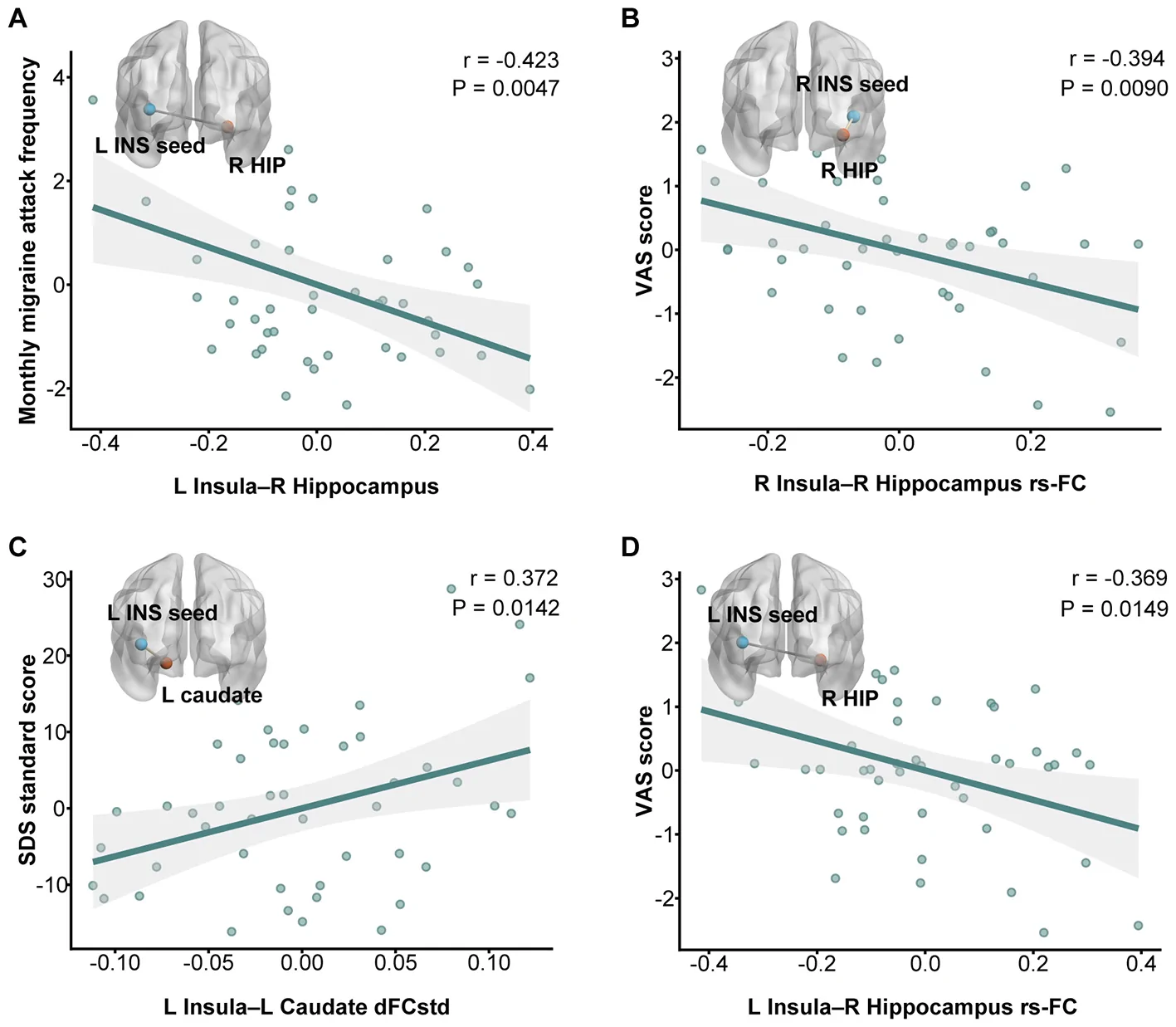
Clinical correlations of altered connectivity. **(A)** Left insula–right hippocampus rs-FC and monthly migraine attack frequency. **(B)** Right insula–right hippocampus rs-FC and VAS score. **(C)** Left insula–left caudate dFCstd and SDS standard score. **(D)** Left insula–right hippocampus rs-FC and VAS score. Scatterplots show age- and sex-adjusted partial correlations in patients with MwoA; shaded areas indicate 95% confidence intervals. *P-*values are uncorrected. MwoA, migraine without aura; rs-FC, resting-state functional connectivity; dFCstd, standard deviation of dynamic functional connectivity; VAS, visual analog scale; SDS, Self-Rating Depression Scale.

Within the MwoA group, partial correlations adjusted for age and sex were used to assess the clinical relevance of the altered connectivity measures. Greater rs-FC linking the left insula to the right hippocampus corresponded to lower monthly migraine attack frequency (*r* = −0.423, *P* = 0.005). Greater rs-FC linking the right insula to the right hippocampus was related to lower VAS scores (*r* = −0.394, *P* = 0.009). In addition, greater dFCstd of the left insular seed with the ipsilateral caudate nucleus corresponded to higher SDS standard scores (*r* = 0.372, *P* = 0.014), whereas rs-FC linking the left insula to the right hippocampus also showed an inverse relationship with VAS score (*r* = −0.369, *P* = 0.015). All reported *P*-values were uncorrected. None of these correlations survived FDR correction. Therefore, they should be interpreted as exploratory findings. These correlation results are displayed in Figure 4.

### 3.5 Sensitivity analyses

Because group differences were observed in SAS and SDS standard scores, additional analyses were performed with SAS and SDS standard scores included as covariates. After further adjustment for anxiety and depressive symptoms, the group differences in the four main altered connections remained significant after FDR correction. Detailed results are presented in Table 4.

**Table 4:** Sensitivity analysis of the main altered connections after additional adjustment for SAS and SDS standard scores.

Metric	Seed	Target	Group β	t-value	*P*	FDR-corrected *P*	Direction
rs-FC	Insula_L	Hippocampus_R	0.218	4.875	< 0.001	< 0.001	MwoA > HCs
rs-FC	Insula_R	Hippocampus_R	0.211	4.722	< 0.001	< 0.001	MwoA > HCs
dFCstd	Insula_L	Caudate_L	−0.067	−4.184	< 0.001	< 0.001	MwoA < HCs
dFCstd	Insula_L	Frontal_Sup_Orb_L	0.049	3.059	0.003	0.003	MwoA > HCs

Insula_L, left insula; Insula_R, right insula; Hippocampus_R, right hippocampus; Caudate_L, left caudate nucleus; Frontal_Sup_Orb_L, left superior frontal gyrus, orbital part; rs-FC, resting-state functional connectivity; dFCstd, standard deviation of dynamic functional connectivity; MwoA, migraine without aura; HCs, healthy controls; FDR, false discovery rate.

After additional adjustment for mean FD, the group differences in all four main altered connections remained significant after FDR correction, with unchanged effect directions (all FDR-corrected *P* < 0.001; Supplementary Table S1). These findings supported the robustness of the main results to residual head motion.

All four main altered connections also remained significant after a more conservative joint FDR correction across all 460 bilateral insula rs-FC and dFCstd comparisons, indicating that the main findings were not dependent on the initial seed-level correction strategy. Detailed results are presented in Supplementary Table S2.

The dFCstd sliding-window sensitivity analysis showed that the two significant dFCstd findings from the primary analysis had generally consistent effect directions under the 30 TR and 60 TR windows. The dFCstd effect linking the left insular seed to the ipsilateral caudate nucleus survived FDR correction under both alternative window lengths. The dFCstd effect involving the left insular seed and the orbital part of the left superior frontal gyrus showed the same direction of change but did not survive FDR correction under either alternative window length. The results are presented in Supplementary Table S3.

### 3.6 Exploratory classification analyses

**Figure 5:**
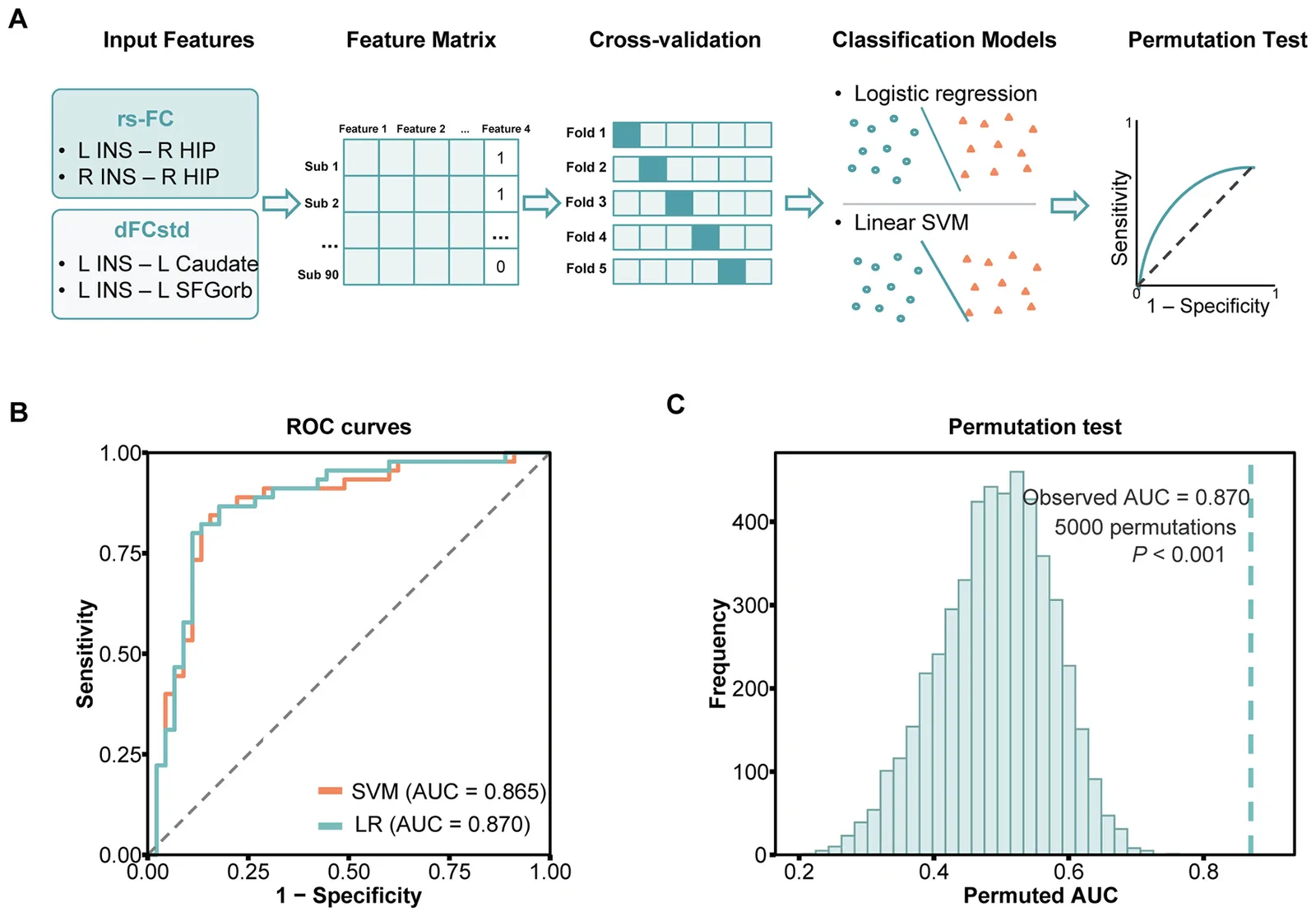
Exploratory classification analysis. **(A)** Workflow of the exploratory classification analysis. **(B)** ROC curves of the logistic regression and linear SVM models. **(C)** Permutation test of the logistic regression model. Models were based on the four altered insula-related connectivity features and evaluated using stratified 5-fold cross-validation. MwoA, migraine without aura; HCs, healthy controls; L, left; R, right; INS, insula; HIP, hippocampus; SFGorb, superior frontal gyrus, orbital part; rs-FC, resting-state functional connectivity; dFCstd, standard deviation of dynamic functional connectivity; ROC, receiver operating characteristic; AUC, area under the curve; SVM, support vector machine; LR, logistic regression.

Exploratory classification analyses based on the four main altered connections showed that both logistic regression and linear support vector machine models distinguished patients with MwoA from HCs in the current sample, with AUCs of 0.870 and 0.865, respectively. Across 20 repeated stratified fivefold cross-validation runs, the mean AUCs were 0.880 ± 0.008 for logistic regression and 0.881 ± 0.007 for linear SVM, showing generally consistent discriminative performance across different random data partitions. Permutation testing indicated that both models performed above chance level (*P* < 0.001). Figure 5 summarizes the exploratory classification workflow and model performance. Supplementary Table S4 contains detailed classification metrics.

In the sensitivity classification analysis using all candidate connections, both logistic regression and linear support vector machine models still showed discriminative performance after feature selection was completed within each training fold, with AUCs of 0.795 and 0.818, respectively. Permutation testing confirmed that both models performed above chance level (both *P* = 0.001). Supplementary Table S5 reports the full results of this sensitivity classification analysis.

## 4 Discussion

Seed-based analyses were conducted using the bilateral insulae as regions of interest to characterize both static rs-FC and the temporal variability of functional connectivity, as measured by dFCstd. The MwoA group exhibited stronger rs-FC between both insular seeds and the right hippocampus than HCs. dFCstd was reduced between the left insula and the left caudate nucleus but increased between the left insula and the orbital part of the left superior frontal gyrus. Exploratory partial correlation analyses further suggested preliminary associations between several altered connectivity measures and monthly migraine attack frequency, pain intensity, or depressive symptoms. These findings suggest that insula-centered connectivity alterations in MwoA involve stronger average connectivity with the right hippocampus and altered temporal variability in connections with the left caudate nucleus and the orbital part of the left superior frontal gyrus. These abnormalities may reflect altered functional coupling among regions implicated in interoceptive pain processing, memory and emotional processing, striatal regulation, and orbitofrontal appraisal and cognitive control.

### 4.1 Insula–hippocampal rs-FC

In the static analysis, rs-FC linking each insular seed to the right hippocampus was elevated in the MwoA group relative to HCs, suggesting stronger average functional coupling between these regions. The insula is known to integrate sensory, affective, cognitive, and autonomic information and has been regarded as a key region in migraine-related brain networks (18, 25). Recent studies focusing on insular subregions in MwoA have further reported structural and functional connectivity alterations, some of which are associated with clinical features such as headache intensity, attack frequency, and disease duration (19, 26).

As a key limbic structure, the hippocampus participates in episodic memory, emotional regulation, stress responses, and the processing of pain-related memories (27, 28). Neuroimaging studies in patients with MwoA have identified abnormal effective connectivity linking the hippocampus with regions involved in sensory and cognitive dimensions of pain, supporting the possible contribution of the hippocampus to migraine pain processing and symptom generation (29). Further work has also identified altered hippocampal connectivity dynamics and hippocampal subfield volumes in migraine, with these hippocampus-related changes being linked to cognitive dysfunction (30). Together, the elevated rs-FC of both insular seeds with the right hippocampus may reflect stronger coupling between regions involved in interoceptive pain processing and limbic emotion and memory systems, which may be relevant to pain-related memory, symptom anticipation, and emotional arousal.

Exploratory partial correlation analyses indicated that stronger rs-FC linking the left insula to the right hippocampus was nominally associated with fewer migraine attacks per month and lower VAS scores in the MwoA group. A similar inverse association with VAS score was observed for rs-FC linking the right insula to the right hippocampus. Although these correlations did not survive FDR correction, they suggest possible relationships between insula–hippocampal functional coupling and headache characteristics or pain intensity. However, their clinical significance remains uncertain.

### 4.2 Insula–caudate dFCstd

For dynamic connectivity, patients with MwoA showed significantly lower dFCstd between the left insula and the left caudate nucleus than HCs, suggesting reduced temporal variability in this connection during resting-state scanning. The caudate nucleus forms part of the striatum and contributes to motor regulation, reward processing, cognitive control, and action selection (31, 32). Evidence from pain neuroimaging research further links caudate abnormalities to pain states and functional impairment related to pain (33, 34). In chronic migraine, rs-fMRI studies have identified altered functional connectivity involving the caudate, supporting the potential contribution of this region to the cognitive, sensory, and emotional integration of pain information in migraine (35). This reduced temporal variability indicates that connectivity between the insula and caudate nucleus fluctuated within a narrower range across time windows rather than showing reduced average connectivity strength. This narrower dynamic range may reflect more constrained reconfiguration of insula–caudate coupling across changing internal or pain-related states.

dFC analysis may capture fluctuations in functional connectivity that are not fully detected by conventional static connectivity analyses, and may help identify migraine-related connectivity abnormalities that are not evident in static analyses (36–38). Evidence from chronic pain research further indicates that pain conditions may involve disrupted functional coupling and abnormal temporal variability across pain processing networks (39).

Exploratory correlation analysis indicated that greater dFCstd of the left insular seed with the ipsilateral caudate nucleus corresponded to higher SDS standard scores in the MwoA group, suggesting that variability in this dynamic connection may be related to depressive symptoms. Emotional factors, including anxiety and depression, have been closely linked to migraine chronification and symptom burden (40, 41). This association indicates that altered dynamic connectivity between the insula and striatum may be relevant to emotional symptoms in migraine. Future longitudinal studies or analyses stratified by emotional symptom severity may help clarify the relationship between insula-related striatal dynamic connectivity and emotional comorbidity in migraine.

### 4.3 Insula–superior frontal dFCstd

By contrast, patients with MwoA showed greater dFCstd between the left insula and the orbital part of the left superior frontal gyrus, suggesting increased temporal variability in this connection during resting-state scanning. Orbitofrontal regions more broadly have been implicated in reward evaluation, emotional decision making, pain anticipation, and cognitive regulation. Evidence from cognitive and affective neuroscience indicates that these regions contribute to the encoding of emotional and reward value, as well as to decision processes involving reward, punishment, and affective experience (42, 43). The insula contributes to interoceptive monitoring, pain perception, and the integration of emotional experience, whereas orbitofrontal regions contribute to the evaluation of pain and its emotional value. Thus, increased dynamic variability in this connection reflects larger window-to-window fluctuations in the coupling between the insula and the orbital part of the superior frontal gyrus.

This increase contrasted with the lower dFCstd observed for the left insular seed and ipsilateral caudate nucleus, indicating that insula-related dynamic alterations in MwoA do not follow a single direction of change. Specifically, reduced variability between the insula and caudate nucleus may reflect a narrower range of dynamic reconfiguration, potentially limiting flexible adjustment of functional coupling across changing internal states. In contrast, increased variability between the insula and the orbital part of the superior frontal gyrus may reflect greater fluctuations and less consistent coupling between interoceptive monitoring and orbitofrontal appraisal or regulatory processes. A recent longitudinal functional connectivity study showed that brain connectivity in migraine varies across the migraine cycle, with connectivity between the anterior insula and posterior orbitofrontal cortex being strongest during the preictal phase and decreasing during the headache phase (9). This longitudinal evidence broadly supports the involvement of interactions between the insula and orbitofrontal regions in brain state changes associated with migraine, providing biological plausibility for the dFCstd abnormality observed here.

### 4.4 Complementary information from rs-FC and dFCstd

rs-FC and dFCstd characterize different aspects of functional connectivity. rs-FC mainly quantifies the average strength of connectivity across the entire scan, whereas dFCstd reflects the magnitude of temporal fluctuations in connectivity and may capture dynamic features that are not evident in static analyses (44). A study of vestibular migraine reported abnormalities in both temporal dynamics and regional concordance of brain function, suggesting that dynamic metrics can provide information complementary to static measures (45). Because the abnormal connections identified by rs-FC and dFCstd did not completely overlap, the two measures appear to capture distinct dimensions of insula-centered functional connectivity in MwoA, including differences in average coupling strength and in the temporal stability or flexibility of connectivity.

### 4.5 Robustness and exploratory discriminative value

The generally consistent findings across sensitivity analyses strengthen confidence in the primary imaging results. After additional adjustment for SAS and SDS standard scores, the main differences in functional connectivity related to the insula remained significant, suggesting that these connectivity abnormalities were not fully explained by group differences in anxiety and depressive symptoms. The joint FDR correction across all bilateral insula rs-FC and dFCstd comparisons further supported the robustness of the main findings. Although the primary analyses used seed-level FDR correction based on the a priori seed design, all four altered connections remained significant under joint correction, suggesting that the findings were not dependent on the initial correction scope.

Methodological studies have noted that estimates from sliding window based dFC analyses may be affected by parameters such as window length (46, 47). In the present study, sensitivity analyses using different window lengths further suggested that the dynamic connectivity abnormality between the insula and caudate nucleus was relatively stable, whereas the finding involving the orbital part of the left superior frontal gyrus appeared more sensitive to window parameters. This difference suggests that insula related dynamic connectivity abnormalities may vary in their robustness in MwoA, and should be further examined in larger samples and under different parameter settings.

Exploratory classification analyses showed that insula-related rs-FC and dFCstd features provided preliminary discrimination between patients with MwoA and HCs in the current sample, suggesting potential discriminative value of these connectivity abnormalities. Methodological work in neuroimaging based classification has emphasized the need to avoid bias from feature selection and to interpret prediction results from small samples with caution (48, 49). The sensitivity classification analysis based on all candidate connections yielded broadly similar discriminative performance, suggesting that connectivity features related to the insula may retain discriminative potential across different analytic strategies. This finding also provides additional but preliminary support for the relevance of insula related functional connectivity abnormalities to brain network dysfunction in MwoA. Given the modest sample size relative to the high-dimensional candidate feature space, overfitting cannot be excluded despite restricting feature selection and preprocessing to the training folds. These classification analyses were exploratory and hypothesis-generating and were not intended for biomarker development. The reported classification performance should therefore be interpreted cautiously and requires independent external validation.

### 4.6 Limitations

Several limitations should be acknowledged. First, because this study used a cross-sectional case-control design, causal links cannot be inferred between altered functional connectivity involving the insula and the onset or progression of MwoA. Future longitudinal research is required to address this question. Second, the present analysis was performed at the ROI level using the AAL116 atlas, with the bilateral insulae treated as whole seed regions. We did not further distinguish subregions of the insula, hippocampus, caudate nucleus, or the orbital part of the left superior frontal gyrus, which may have reduced the sensitivity for detecting more fine-grained connectivity alterations and may have obscured distinct or opposing changes across insular subregions. Future studies using more refined brain parcellations may help characterize these patterns in greater detail. Third, headache laterality was not systematically recorded, preventing us from determining whether connectivity alterations involving the left and right insulae were associated with ipsilateral or contralateral headache symptoms. Future studies should prospectively record headache laterality and compare adequately sized groups with headache affecting the left side, the right side, alternating sides, or both sides. Fourth, the present cohort had a relatively restricted range of monthly migraine attack frequency, and monthly migraine days (MMD) and acute medication days (AMD) were not systematically recorded. This limited a more complete characterization of baseline migraine burden and prevented formal classification into low-frequency and high-frequency episodic migraine subgroups. Future studies should prospectively record both MMD and AMD and prespecify subgroup analyses according to migraine frequency. Fifth, the relatively small sample size may limit the stability and generalizability of the clinical association results, which require validation in larger samples. Finally, although the classification analyses suggested that connectivity features involving the insula showed preliminary discriminative ability, the study was based on a single-center sample and lacked independent external validation. Thus, the correlation and classification findings should be viewed as preliminary evidence of clinical relevance and potential discriminative value, respectively. Both require further validation in larger, multicenter, or longitudinal studies.

## 5 Conclusion

Using bilateral insulae as seed regions in rs-fMRI, this study identified abnormalities in both static and dynamic functional connectivity in patients with MwoA. These abnormalities were mainly characterized by higher rs-FC between the bilateral insulae and the right hippocampus, lower dFCstd between the left insula and the left caudate nucleus, and higher dFCstd between the left insula and the orbital part of the left superior frontal gyrus. These findings suggest that MwoA may involve altered insula-centered connectivity with the right hippocampus, left caudate nucleus, and orbital part of the left superior frontal gyrus. Combining rs-FC and dFCstd may provide complementary information by capturing both average connectivity strength and temporal variability, thereby providing a more comprehensive characterization of insula-centered functional connectivity abnormalities in MwoA. Exploratory classification analyses further suggest that these connectivity features may have preliminary discriminative value, although validation in larger and independent datasets is still needed.

## Data Availability

The raw data supporting the conclusions of this article will be made available by the authors, without undue reservation.
