# Supplementary material for: Static and dynamic functional connectivity of the bilateral insula in patients with migraine without aura: a resting-state fMRI study
Source: Front Neurol. 2026 Aug 31;17:1898658. doi: 10.3389/fneur.2026.1898658 (PMC13572113; doi:10.3389/fneur.2026.1898658)
Supplement: Supplementary file 1 [file Table_1.docx]

***Supplementary Material***

**Contents**

Supplementary Methods 1. Mean FD covariate sensitivity analysis

Supplementary Methods 2. Joint FDR sensitivity analysis

Supplementary Methods 3. Sliding-window length sensitivity analysis

Supplementary Methods 4. Exploratory machine learning classification analysis

Supplementary Results 1. Mean FD covariate sensitivity analysis

Supplementary Results 2. Joint FDR correction

Supplementary Results 3. Sliding-window length sensitivity analysis

Supplementary Results 4. Exploratory machine learning classification analysis

Supplementary Table S1. Sensitivity analysis of the main altered connections after additional adjustment for mean framewise displacement.

Supplementary Table S2. Joint FDR correction across bilateral insula rs-FC and dFCstd analyses.

Supplementary Table S3. Sensitivity analysis of dFCstd using different sliding-window lengths.

Supplementary Table S4. Exploratory classification analysis based on four main altered connections.

Supplementary Table S5. Sensitivity classification using all candidate connections as the feature pool.

Supplementary Table S6. Frequently selected features across cross-validation folds.

Supplementary Figure S1. Sensitivity classification using all candidate connections as the feature pool.

# 1 **Supplementary Methods**

## 1.1 Mean FD covariate sensitivity analysis

## To further assess the potential influence of residual head motion on the main findings, we performed an additional sensitivity analysis for the four connections that survived FDR correction in the primary analyses. For each connection, a general linear model was constructed with group as the independent variable and age, sex, and mean framewise displacement calculated using the Power method as covariates. FDR correction was applied across the group effects of the four connections.

## 1.2 **Joint FDR sensitivity analysis**

To assess the robustness of the main findings to the multiple-comparison correction strategy, we performed a joint FDR sensitivity analysis. All seed-to-target connections from the bilateral insula rs-FC and dFCstd analyses were entered into a single FDR correction framework. The joint correction included 460 tests in total, comprising 115 connections for each of the left insula rs-FC, right insula rs-FC, left insula dFCstd, and right insula dFCstd analyses. The group-comparison models and covariates were identical to those used in the main analysis. Connections that remained significant after joint FDR correction were considered robust under a more stringent multiple-comparison control strategy.

## 1.3 **Sliding-window length sensitivity analysis**

To assess the sensitivity of the dFCstd findings to the sliding-window length, we recalculated bilateral insula seed-to-target dFCstd using 30 TR and 60 TR windows, in addition to the 50 TR window used in the main analysis. All other parameters, including the step size, were kept unchanged. Within each sliding window, Pearson correlations were calculated between the time series of each insula seed and each target region and were then Fisher z-transformed. For each connection, dFCstd was defined as the standard deviation of Fisher z values across all windows. The group-comparison model was identical to that used in the main analysis. We then compared the effect direction and FDR-corrected significance of the main FDR-significant dFCstd connections across window lengths to evaluate the robustness of these findings to window-length selection.

## 1.4 **Exploratory machine learning classification analysis**

To further evaluate whether insula-related connectivity features could distinguish patients with MwoA from HCs, we performed post hoc exploratory machine learning classification analyses. First, the four insula-related connections that survived FDR correction in the main analysis were used as input features to train logistic regression and linear support vector machine models. The analyses were conducted in R using the caret, e1071, and pROC packages. Logistic regression was implemented as an unpenalized binomial generalized linear model, with a predicted probability threshold of 0.5 used for classification. The linear SVM used a linear kernel with a fixed cost parameter of 1.0 and probability estimation enabled. No class weighting or hyperparameter tuning was applied, and other parameters were retained at their default settings. Model performance was evaluated using stratified fivefold cross-validation. To further assess the sensitivity of model performance to random data partitioning, the stratified fivefold cross-validation procedure was additionally repeated 20 times using different random partitions while preserving the class distribution within each fold. AUC, accuracy, sensitivity, specificity, and balanced accuracy were calculated for each repetition and summarized across the 20 repetitions as mean and standard deviation. Within each fold, feature-standardization parameters were estimated from the training data and subsequently applied to the corresponding test data. Permutation testing with 5000 repetitions was used to determine whether classification performance exceeded chance level, with the complete cross-validation procedure repeated for both models in each permutation. This analysis was considered exploratory because the input features were derived from the main group comparison results. Detailed results are presented in the main text and Supplementary Table S4.

To reduce the potential bias introduced by selecting features from significant findings in the full sample, we further conducted a stricter sensitivity classification analysis using all candidate connections as the feature pool. Candidate features included rs-FC and dFCstd connections between the bilateral insula seeds and the remaining 115 AAL116 target regions, yielding 460 connectivity features in total. During stratified fivefold cross-validation, age and sex residualization, selection of the top 10 features, feature scaling, and model training were all performed within each training fold. Candidate features were ranked according to the absolute two-sample t statistic calculated within each training fold. The corresponding test fold was not used for feature selection, estimation of scaling parameters, or model fitting. The same logistic regression and linear SVM settings described above were used. Permutation testing repeated the full cross-validation procedure 1000 times to assess whether model performance exceeded chance level.

# 2 **Supplementary Results**

## 2.1 Mean FD covariate sensitivity analysis

## After additional adjustment for mean FD, the group differences in all four main altered connections remained significant after FDR correction, with unchanged effect directions (all FDR-corrected *P* < 0.001). These results indicate that the main findings were robust to residual variation in head motion, as shown in Supplementary Table S1.

## 2.2 **Joint FDR correction**

The joint FDR correction showed that all four connections identified in the main analysis remained significant after correction across 460 tests. These connections included rs-FC between the left insula and the right hippocampus, rs-FC between the right insula and the right hippocampus, dFCstd between the left insula and the left caudate nucleus, and dFCstd between the left insula and the left superior frontal gyrus, orbital part. These findings suggest that the main group differences remained stable under a more conservative multiple-comparison correction strategy, as shown in Supplementary Table S2.

## 2.3 **Sliding-window length sensitivity analysis**

The sliding-window length sensitivity analysis showed that the two dFCstd connections that survived FDR correction in the main analysis had the same effect direction when 30 TR and 60 TR windows were used. The dFCstd between the left insula and the left caudate nucleus remained significant after FDR correction under both alternative window lengths. In contrast, the dFCstd between the left insula and the left superior frontal gyrus, orbital part showed a consistent direction, but did not remain significant after FDR correction under the alternative window lengths. These results suggest that the dFCstd change between the left insula and the left caudate nucleus was relatively robust to window-length selection, whereas the finding for the left insula and the left superior frontal gyrus, orbital part may be more sensitive to this parameter and should be interpreted with caution, as shown in Supplementary Table S3.

## 2.4 **Exploratory machine learning classification analysis**

The post hoc exploratory classification analysis based on the four main differential connections showed that both logistic regression and linear support vector machine models could distinguish patients with MwoA from HCs in the current sample, with AUC values of 0.870 and 0.865, respectively. The repeated cross-validation analysis showed generally consistent classification performance across different random data partitions. Variability in AUC, accuracy, sensitivity, specificity, and balanced accuracy across the 20 repetitions was limited for both logistic regression and linear SVM. Permutation testing with 5000 repetitions showed that the classification performance of both models exceeded chance level, *P* < 0.001. Detailed classification metrics are provided in Supplementary Table S4.

The sensitivity classification analysis using all 460 insula-related connections as the candidate feature pool also showed discriminative performance, without selecting features from the full sample in advance. The logistic regression and linear support vector machine models achieved AUC values of 0.795 and 0.818, respectively, and permutation testing with 1000 repetitions showed that both models performed above chance level, both *P =* 0.001. Other classification metrics are provided in Supplementary Table S5, and the ROC curves and permutation-test results are shown in Supplementary Figure S1.

In this sensitivity classification analysis, the connections involving the left insula and right hippocampus, right insula and right hippocampus, left insula and left caudate nucleus, and left insula and left superior frontal gyrus, orbital part were repeatedly selected across different cross-validation folds. This pattern was broadly consistent with the main group comparison findings (Supplementary Table S6).

Overall, insula-related static and dynamic functional connectivity features showed preliminary discriminative ability in the current sample. However, because these classification analyses were based on a single-center sample and lacked independent external validation, the findings should be interpreted as exploratory and sensitivity analyses rather than evidence for a diagnostic model or a stable imaging biomarker.

# 3 **Supplementary Tables and Figure**

**Supplementary Table S1.** Sensitivity analysis of the main altered connections with adjustment for mean FD.

| **Metric** | **Seed** | **Target** | **Group β** | **t value** | **FDR *P*** | **Direction** |
| --- | --- | --- | --- | --- | --- | --- |
| rs-FC | Insula_L | Hippocampus_R | 0.183 | 4.513 | <0.001 | MwoA > HCs |
| rs-FC | Insula_R | Hippocampus_R | 0.160 | 3.877 | <0.001 | MwoA > HCs |
| dFCstd | Insula_L | Caudate_L | −0.065 | −4.673 | <0.001 | MwoA < HCs |
| dFCstd | Insula_L | Frontal_Sup_Orb_L | 0.053 | 3.813 | <0.001 | MwoA > HCs |

**Note:** General linear models included group, age, sex, and mean FD calculated using the Power method as covariates. Group β represents the unstandardized group coefficient, with HC coded as 0 and MwoA coded as 1. FDR correction was applied across the four main altered connections. FD, framewise displacement; FDR, false discovery rate; rs-FC, resting-state functional connectivity; dFCstd, standard deviation of dynamic functional connectivity; MwoA, migraine without aura; HCs, healthy controls.

**Supplementary Table S2.** Joint FDR correction across bilateral insula rs-FC and dFCstd analyses.

| **Metric** | **Seed** | **Target** | **Direction** | **t** | **Raw *P*** | **Seed-level FDR *P*** | **Joint FDR *P*** | **Cohen's *d*** |
| --- | --- | --- | --- | --- | --- | --- | --- | --- |
| rs-FC | Insula_L | Hippocampus_R | MwoA > HCs | 4.518 | <0.001 | 0.002 | 0.005 | 0.922 |
| rs-FC | Insula_R | Hippocampus_R | MwoA > HCs | 3.870 | <0.001 | 0.024 | 0.032 | 0.815 |
| dFCstd | Insula_L | Caudate_L | MwoA < HCs | -4.607 | <0.001 | 0.002 | 0.005 | -0.976 |
| dFCstd | Insula_L | Frontal_Sup_Orb_L | MwoA > HCs | 3.776 | <0.001 | 0.017 | 0.034 | 0.730 |

**Note:** Insula_L, left insula; Insula_R, right insula; Hippocampus_R, right hippocampus; Caudate_L, left caudate nucleus; Frontal_Sup_Orb_L, left superior frontal gyrus, orbital part; FDR, false discovery rate.

**Supplementary Table S3.** Sensitivity analysis of dFCstd using different sliding-window lengths.

| **Window length** | **Seed** | **Target** | **Direction** | **FDR *P*** |
| --- | --- | --- | --- | --- |
| 50 TR | Insula_L | Caudate_L | MwoA < HCs | 0.002 |
| 50 TR | Insula_L | Frontal_Sup_Orb_L | MwoA > HCs | 0.017 |
| 30 TR | Insula_L | Caudate_L | MwoA < HCs | 0.002 |
| 30 TR | Insula_L | Frontal_Sup_Orb_L | MwoA > HCs | 0.124 |
| 60 TR | Insula_L | Caudate_L | MwoA < HCs | 0.005 |
| 60 TR | Insula_L | Frontal_Sup_Orb_L | MwoA > HCs | 0.765 |

**Note:** Insula_L, left insula; Caudate_L, left caudate nucleus; Frontal_Sup_Orb_L, left superior frontal gyrus, orbital part; FDR, false discovery rate.

**Supplementary Table S4.** Exploratory classification analysis based on the four main altered connections.

| **Model** | **Validation** | **AUC** | **Accuracy** | **Sensitivity** | **Specificity** | **Balanced accuracy** | **Permutation P** |
| --- | --- | --- | --- | --- | --- | --- | --- |
| Logistic regression | 5-fold CV | 0.870 | 0.822 | 0.867 | 0.778 | 0.822 | <0.001 |
|  | Repeated 5-fold CV (20 repeats) | 0.880 ± 0.008 | 0.837 ± 0.016 | 0.850 ± 0.024 | 0.824 ± 0.031 | 0.837 ± 0.016 | — |
| Linear SVM | 5-fold CV | 0.865 | 0.844 | 0.844 | 0.844 | 0.844 | <0.001 |
|  | Repeated 5-fold CV (20 repeats) | 0.881 ± 0.007 | 0.843 ± 0.015 | 0.849 ± 0.019 | 0.837 ± 0.026 | 0.843 ± 0.015 | — |

**Note:** AUC, area under the receiver operating characteristic curve; SVM, support vector machine. All cross-validation procedures were stratified to preserve the class distribution across folds. Repeated 5-fold cross-validation results are reported as mean ± SD across 20 repetitions. Permutation *P* values were obtained from 5000 permutations based on the original 5-fold cross-validation.

**Supplementary Table S5.** Sensitivity classification using all candidate connections as the feature pool.

| **Model** | **Selected features** | **C** | **AUC** | **Accuracy** | **Sensitivity** | **Specificity** | **Balanced accuracy** | **Permutation *P*** |
| --- | --- | --- | --- | --- | --- | --- | --- | --- |
| Logistic regression | 10 | - | 0.795 | 0.756 | 0.756 | 0.756 | 0.756 | 0.001 |
| Linear SVM | 10 | 1.0 | 0.818 | 0.778 | 0.800 | 0.756 | 0.778 | 0.001 |

**Note:** Candidate features included 460 bilateral insula seed-to-target connections. Feature residualization, top-10 selection, scaling, and model fitting were performed within each training fold. Permutation *P* values were based on 1000 permutations.

**Supplementary Table S6.** Frequently selected features across cross-validation folds.

| **Metric** | **Seed** | **Target** | **logistic regression** | **linear SVM** |
| --- | --- | --- | --- | --- |
| rs-FC | Insula_L | Hippocampus_R | 5 / 5 | 5 / 5 |
| dFCstd | Insula_L | Caudate_L | 5 / 5 | 5 / 5 |
| rs-FC | Insula_R | Hippocampus_R | 5 / 5 | 5 / 5 |
| dFCstd | Insula_L | Frontal_Sup_Orb_L | 4 / 5 | 4 / 5 |
| rs-FC | Insula_L | Hippocampus_L | 3 / 5 | 3 / 5 |
| dFCstd | Insula_R | Parietal_Inf_L | 3 / 5 | 3 / 5 |

**Note:** Features selected in at least 3 of the 5 cross-validation folds are shown. Insula_L, left insula; Insula_R, right insula; Hippocampus_R, right hippocampus; Hippocampus_L, left hippocampus; Caudate_L, left caudate nucleus; Frontal_Sup_Orb_L, left superior frontal gyrus, orbital part; Parietal_Inf_L, left inferior parietal gyrus; SVM, support vector machine.

**Supplementary Figure S1.** Sensitivity classification using all candidate connections as the feature pool. (A) ROC curves of logistic regression and linear SVM models. (B, C) Null distributions of AUC from 1000 permutation tests for logistic regression and linear SVM, respectively. Candidate features included 460 bilateral insula seed-to-target connections, with top-10 feature selection performed within each training fold. AUC, area under the curve; dFCstd, standard deviation of dynamic functional connectivity; LR, logistic regression; ROC, receiver operating characteristic; rs-FC, resting-state functional connectivity; SVM, support vector machine.
